# Neuroprotective Effect of 4-Phenylbutyric Acid against Photo-Stress in the Retina

**DOI:** 10.3390/antiox10071147

**Published:** 2021-07-20

**Authors:** Naymel Alejandra Guzmán Mendoza, Kohei Homma, Hideto Osada, Eriko Toda, Norimitsu Ban, Norihiro Nagai, Kazuno Negishi, Kazuo Tsubota, Yoko Ozawa

**Affiliations:** 1 Laboratory of Retinal Cell Biology, Department of Ophthalmology, School of Medicine, Keio University, 35 Shinanomachi, Shinjuku-ku, Tokyo 160-8582, Japan; naymelguzmanm@keio.jp (N.A.G.M.); hommak@keio.jp (K.H.); hosada@a7.keio.jp (H.O.); riko.4267@gmail.com (E.T.); nban@keio.jp (N.B.); nagai@a5.keio.jp (N.N.); 2 Department of Ophthalmology, School of Medicine, Keio University, 35 Shinanomachi, Shinjuku-ku, Tokyo 160-8582, Japan; kazunonegishi@keio.jp (K.N.); tsubota@z3.keio.jp (K.T.); 3 Department of Ophthalmology, St. Luke’s International Hospital, 9-1 Akashi-cho, Chuo-ku, Tokyo 104-8560, Japan; 4 Laboratory of Retinal Cell Biology, St. Luke’s International University, 9-1 Akashi-cho, Chuo-ku, Tokyo 104-8560, Japan

**Keywords:** retina, neuroprotection, endoplasmic reticulum stress, oxidative stress, inflammation, light-induced retinal damage, photoreceptor, visual function, apoptosis, neurodegeneration, blindness

## Abstract

Exposure to excessive visible light causes retinal degeneration and may influence the progression of retinal blinding diseases. However, there are currently no applied treatments. Here, we focused on endoplasmic reticulum (ER) stress, which can cause cellular degeneration and apoptosis in response to stress. We analyzed functional, histological, and molecular changes in the light-exposed retina and the effects of administering an ER-stress inhibitor, 4-phenylbutyric acid (4-PBA), in mice. We found that light-induced visual function impairment related to photoreceptor cell loss and outer segment degeneration were substantially suppressed by 4-PBA administration, following attenuated photoreceptor apoptosis. Induction of retinal ER stress soon after light exposure, represented by upregulation of the immunoglobulin heavy chain binding protein (BiP) and C/EBP-Homologous Protein (CHOP), were suppressed by 4-PBA. Concurrently, light-induced oxidative stress markers, Nuclear factor erythroid 2–related factor 2 (Nrf2) and Heme Oxygenase 1 (HO-1), and mitochondrial apoptotic markers, B-cell lymphoma 2 apoptosis regulator (Bcl-2)-associated death promoter (Bad), and Bcl-2-associated X protein (Bax), were suppressed by 4-PBA administration. Increased expression of glial fibrillary acidic protein denoted retinal neuroinflammation, and inflammatory cytokines were induced after light exposure; however, 4-PBA acted as an anti-inflammatory. Suppression of ER stress by 4-PBA may be a new therapeutic approach to suppress the progression of retinal neurodegeneration and protect visual function against photo-stress.

## 1. Introduction

Exposure to excessive visible light causes retinal degeneration and may influence progression of retinitis pigmentosa (RP) [1,2,3,4] and age-related macular degeneration [5,6,7,8] both of which are leading causes of blindness worldwide [5,7,9,10]. The mechanisms involved in light-induced retinal degeneration are historically documented [8,11]. However, there are no applied treatments for alleviating the progression of retinal disorders caused by photo-stress.

Here, we focused on endoplasmic reticulum (ER) stress, which is induced after light exposure (LE) [12,13,14], as well as oxidative stress [8,12,15,16]. The ER is an interconnected network of branching tubules and flattened sacs and plays a major role in biosynthesis, post-translational modification, folding, and assembly of newly synthesized proteins [17,18,19]. In the presence of misfolded or unfolded proteins in the ER lumen, the unfolded protein response (UPR) induces transcriptional and translational events to restore ER homeostasis. However, when the capacity of the ER is overwhelmed, apoptotic signaling is activated leading to cell death [17,18,19,20]. ER stress is involved in various human diseases, such as diabetes, cancer, and neurodegeneration [17,18,19,20,21,22,23]. ER stress has a close relationship with oxidative stress and inflammation in neurodegeneration and retinal diseases [12,13,14,17,18,19,20,21,22]. However, whether ER stress has a critical role and could be a therapeutic target against the influences of photo-stress have not been determined, and no methods or pharmaceuticals have been developed for its management.

4-phenylbutyric acid (4-PBA), is an aromatic fatty acid that has being approved by the United States Food and Drug Administration (FDA) for the treatment of urea cycle disorders (UCDs) for its property as an ammonia scavenger, while it also acts as a chemical chaperone that inhibits ER stress [24]. 4-PBA has also been shown to affect various biological functions including adipogenesis, autophagy, inflammation, and glucose homeostasis [24,25,26,27,28,29]; it has also been demonstrated to rescue oligodendrocytes under conditions of hypoxia such as after nonarteritic anterior ischemic optic neuropathy (AION) [30] and normobaric hypoxia model using an hypoxia chamber [31], to offer neuroprotection for retinal ganglion cells (RGCs) against high intraocular pressure-induced optic nerve ischemia [32], to suppress the elevation of endothelial permeability and leakage of extracellular-superoxide dismutase in proliferative diabetic retinopathy [33] and to improve cone cell survival in Leber congenital amaurosis (LCA) [34]. Thus, to assess whether 4-PBA suppresses ER stress in the light-exposed retina and whether it leads to suppressing subsequent pathological reactions and cell death would be of value to propose a new therapeutic approach for retinal photo-stress.

In this study, we investigated the effect of 4-PBA administration in a light-induced retinal degeneration model in mice to determine the contribution of ER stress in retinal neurodegeneration and its relationships between oxidative stress and inflammation. This study will help propose a new therapeutic approach for photo-stress-related diseases.

## 2. Materials and Methods

### 2.1. Animals

Eight-to-nine-week-old male BALB/c mice (CLEA Japan, Tokyo, Japan) were housed in an air-conditioned room maintained at 22 ± 2 °C under a 12-h dark/light cycle (light on from 8:00 to 20:00), with dim white light (25 lux) and free access to a standard diet (CLEA Japan, Tokyo, Japan) and tap water. The mice were randomly divided into three groups: (1) control group (no LE with vehicle treatment), (2) LE-with-vehicle-treatment group and (3) LE-with-4-PBA-treatment group. All animal experiments were designed to comply with the Animal Research: Reporting of In Vivo Experiments (ARRIVE) guidelines and were performed in accordance with the Association for Research in Vision and Ophthalmology (ARVO) Statement for the Use of Animals in Ophthalmic and Vision Research, the National Institutes of Health guide for the care, and the guidelines for the Animal Care Committee of Keio University (Approval Number, 08002).

### 2.2. Light-Induced Retinal Degeneration Model and Administration of 4-PBA

All mice were dark-adapted by maintaining them in complete darkness overnight prior to LE. Their pupils were dilated with a mixture of 0.5% tropicamide and 0.5% phenylephrine (Mydrin-P^®^; Santen Pharmaceutical, Osaka, Japan) just before exposure to light. Subsequently, the LE-with-4-PBA-treatment group was administered 4-PBA solution (4-PBA; Sigma Aldrich, St. Louis, MO, USA) dissolved in filtered phosphate-buffered saline (PBS) via intraperitoneal injection at a single dose of 30 mg/kg. In the control and LE-with-vehicle-treatment groups, mice received a volume-matched intraperitoneal injection of filtered PBS. Immediately after injection, mice (excluding control group mice) were exposed to 2500 lux from a white fluorescent lamp (FHD100ECW; Panasonic, Osaka, Japan) for 1 h, in a dedicated exposure box maintained at 22 ± 2 °C, containing stainless-steel mirrors on all walls and the floor (Tinker N, Kyoto, Japan). Following LE, or for the control mice following vehicle injection, the mice were returned to their cages and maintained under the previously mentioned dim cyclic light until they were euthanized with an anesthetic mixture of medetomidine hydrochloride (Domitor^®^ Nippon Zenyaku Kogyo Co., Ltd., Tokyo, Japan), midazolam (Midazolam Sandoz^®^, Sandoz, Tokyo, Japan), and butorphanol (Vetorphale^®^, Meiji Seika Pharma, Tokyo, Japan), diluted with filtered PBS and mixed at doses of 0.75, 4.0, and 5.0 mg/kg body weight, respectively, followed by cervical dislocation at different sampling time points according to the experimental protocol.

### 2.3. Electroretinogram (ERG)

Mice were dark-adapted overnight for more than 12 h and then placed under dim-red illumination before conducting the ERGs as previously reported [35,36,37]. Mice were anesthetized with intraperitoneal injection of the anesthetic mixture described above in Section 2.2. Mouse pupils were dilated using a single drop of a mixture of 0.5% tropicamide and 0.5% phenylephrine (Mydrin-P^®^; Santen Pharmaceutical, Osaka, Japan). The ground and reference electrodes were then placed on the tail and in the mouth, respectively, and the active gold wire electrodes were placed on the right eye cornea. Full-field scotopic ERGs were recorded in response to a flash stimulus at intensities ranging from –2.1 to 2.9 log cd s/m^2^ using a PowerLab System 2/25 (AD Instruments, Sydney, New South Wales, Australia). The responses were differentially amplified and filtered through a digital bandpass filter ranging from 0.3 to 1000 Hz. Each stimulus was delivered using a commercially available stimulator (Ganzfeld System SG-2002; LKC Technologies, Inc., Gaithersburg, MD, USA). The a-wave amplitude was measured from the baseline to the negative trough, whereas the b-wave amplitude was measured from the trough of the a-wave to the peak of the b-wave. The implicit times were measured from the onset of the stimulus to the peak of each wave. The peak points were automatically indicated by the system and confirmed by the examiner. The experiments were performed 4 days after LE.

### 2.4. Immunohistochemistry (IHC)

IHC for rhodopsin was performed 4 days after LE. Following ERG, eyes were enucleated and embedded in Optimal Cutting Temperature compound (OCT compound) (Sakura Finetek, Tokyo, Japan) and immediately snap frozen in liquid nitrogen. Cryosections (10 μm) were prepared and fixed with 4% paraformaldehyde (4% PFA) in PBS, permeabilized with 0.1% Triton-X100 in PBS, and then blocked with 5% Bovine Serum Albumin (BSA) in PBS for 1 h at room temperature (RT). Then, the sections were incubated with mouse anti-rhodopsin antibody (1:100,000, catalog # MA1-722, Thermo Fisher Scientific Inc., Waltham, MA, USA) at 4 °C overnight, followed by incubation with Alexa 488-conjugated goat anti-mouse IgG (Invitrogen Japan, Tokyo, Japan) and DAPI (4’,6-diamidino-2-phenylindole) solution (Sigma-Aldrich, St. Louis, MO, USA) at 2 μg/mL for 2 h at RT.

IHC for glial fibrillary acidic protein (GFAP) was performed 24 h after LE. Eyes were sampled and fixed in 4% PFA overnight at 4 °C, and then embedded in paraffin (Sakura Finetek, Tokyo, Japan). Sections (7–8 μm) were deparaffinized by passing them through the following treatment series for 5 min each: xylene, 100% ethanol, 90% ethanol, 70% ethanol, and 50% ethanol, finishing by rinsing with ultrapure water. After, antigen retrieval was performed by heating the sections in Immunosaver solution (1:200; Wako, Tokyo, Japan) for 45 min at 90 °C followed by blocking with 5% BSA in PBS for 1 h at RT, the sections were incubated with rabbit anti-GFAP antibody (1:1,000; Dako, Agilent Pathology Solutions, Santa Clara, CA, USA) at 4 °C overnight. Then, the sections were incubated with Alexa 546-conjugated goat anti-rabbit IgG (Invitrogen Japan, Tokyo, Japan) and DAPI solution DAPI (4’,6-diamidino-2-phenylindole) (Sigma-Aldrich, St. Louis, MO, USA) at 2 μg/mL for 2 h at RT.

Sections, which included the optic nerve head and the most peripheral region of the retina, were examined using a confocal microscope (TCS-SP5; Leica, Tokyo, Japan), and the images were obtained with a digital camera (Olympus Co., Tokyo, Japan). The outer nuclear layer (ONL) thickness was measured from the top to the bottom of the ONL, and the rod photoreceptor outer segment (OS) length was measured from the top to the bottom of OS referring to the rhodopsin staining, both at 500 μm distance from the optic nerve head in the superior central retina using ImageJ (National Institutes of Health, Bethesda, MD, USA; available at http://rsb.info.nih.gov/ij/index.html (accessed 18 June 2021) and averaged as described previously [35,38].

### 2.5. Terminal Deoxynucleotidyl Transferase 2′-Deoxyuridine, 5′-Triphosphate (dUTP) Nick End Labeling (TUNEL) Assay

Eyes were enucleated at 48 h after LE and embedded in OCT compound (Sakura Finetek, Tokyo, Japan) and immediately snap frozen in liquid nitrogen and cryosections (10 μm), including the optic nerve head and the most peripheral region of the retina, were prepared. The TUNEL assay was performed using the ApopTag red apoptosis detection kit (Millipore, Bedford, MA, USA) according to the manufacturer’s protocol, and the nuclei were counterstained with DAPI solution (2 μg/mL; Sigma-Aldrich, St. Louis, MO, USA). Sections were examined, and the images were observed as described above. The TUNEL-positive cells were counted per section and averaged as described previously [12,35,38].

### 2.6. Real-Time, Reverse-Transcription Polymerase Chain Reaction (RT-PCR)

Total RNA was isolated from the retina with TRIzole (Invitrogen, Waltham, MA, USA) either immediately after (0 h) or 3 h, 6 h, or 24 h after LE, and the RNA concentration was measured using a NanoDrop 1000 (Thermo Fisher Scientific, Inc., Waltham, MA, USA) and 1 μg of total RNA was reverse-transcribed using SuperScript VILO master mix (Thermo Fisher Scientific, Inc., Waltham, MA, USA) according to the manufacturer’s instructions. Real-time PCR was performed using the StepOnePlus™ PCR system (Applied Biosystems, Foster City, CA, USA), and gene expression was quantified using the ΔΔCT method. The respective forward and reverse primer sequences used in the study with SYBR system (SYBR™ Green Master Mix, Applied Biosystems™, Waltham, MA, USA) were normalized to 18 s mRNA levels (Table 1). For mRNA levels of B-cell lymphoma 2 apoptosis regulator (Bcl-2)-associated X protein (Bax) and Bcl-2, the TaqMan gene expression assay was used, (Bax, TaqMan probe assay ID; Mm00432050_m1; GenBank Accession No. NM_007527; Thermo Fisher Scientific, Inc., Waltham, MA, USA; Bcl-2 TaqMan probe assay ID; Mm00477631_m1; GenBank Accession No. NM_009741; Thermo Fisher Scientific, Inc., Waltham, MA, USA). Glyceraldehyde-3-Phosphate Dehydrogenase (GAPDH) was used as an endogenous control (AB4351309, Applied Biosystems TM Waltham, MA, USA; GenBank Accession No. NM_001289726).

### 2.7. Enzyme-Linked Immunosorbent Assay (ELISA)

Total protein was isolated from mouse retinas 3 h after LE using lysis buffer that included a protease inhibitor cocktail (Complete, Ethylenediaminetetraacetic acid (EDTA)-free; Roche, Mannheim, Germany) and phosphatase inhibitor cocktails 2 and 3 (Sigma-Aldrich, St. Louis, MO, USA). The immunoglobulin heavy chain binding protein (BiP), also known as 78-kDa glucose regulated protein (GRP78), was measured using ELISA (GRP78/BiP: Enzo Life Sciences, Farmingdale, New York, NY, USA) and a microplate reader (Cytation™ 5 Cell Imaging Multi-Mode Reader; BioTek Instruments, Winooski, VT, USA). All procedures were performed according to the manufacturers’ instructions.

### 2.8. Statistical Analysis

Data are expressed as mean ± standard deviation. By using commercially available software (IBM SPSS Ver 25; IBM Corp, Armonk, New York, NY, USA), one-way analysis of variance (ANOVA) with Tukey post-hoc tests were performed to assess statistical significance between the groups. Results were considered significant at *p* < 0.05.

## 3. Results

### 3.1. 4-PBA Protected Visual Function against Light Exposure

**Figure 1:**
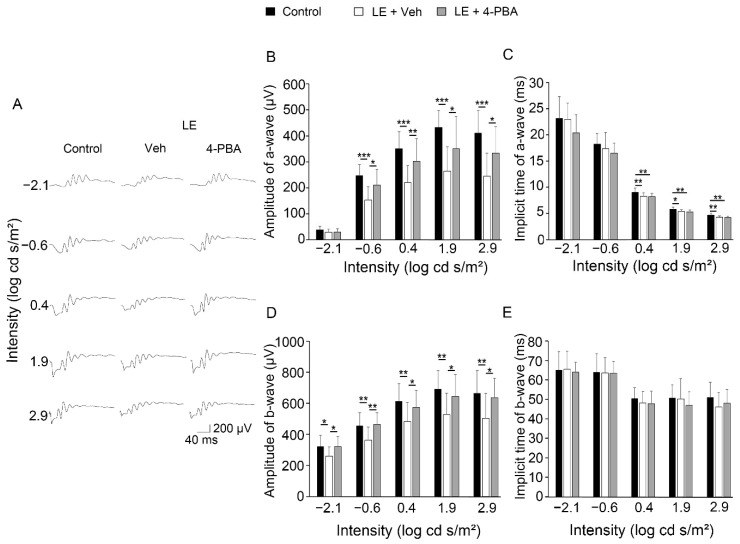
4-phenylbutyric acid (4-PBA) protected visual function against light exposure. (**A**) Representative wave forms of Electroretinogram (ERG) from individual mice at a stimulus intensity from –2.1 to 2.9 log cd s/m^2^ recorded 4 days after light exposure with or without 4-PBA treatment. (**B**–**E**) Mean ± standard deviation of the amplitudes and implicit times of the a-wave (**B**,**C**) and b-wave (**D**,**E**), respectively, at each visual stimulation intensity in each group. Light exposure reduced the amplitudes of the a- and b-waves; however, 4-PBA alleviated this reduction. There were no statistically significant differences between the amplitudes of control and LE-with-4-PBA-treatment-groups. ERG, electroretinogram; LE, light exposure; Veh, vehicle. Control: *n* = 16, LE-with-vehicle-treatment: *n* = 15 and LE-with- 4-PBA treatment: *n* = 15. * *p* < 0.05, ** *p* < 0.01, *** *p* < 0.001. One-way analysis of variance (ANOVA) with Tukey post-hoc tests.

To evaluate the effect of 4-PBA on visual function, we first measured scotopic ERGs 4 days after LE when the photo-stress-induced ERG changes were obvious according to our previous reports (Figure 1A–E) [12,15,35,38,39]. In the ERG, the a-wave reflects the function of the photoreceptors in the ONL, while the b-wave reflects the following electrical actions to the photoreceptor cells. We found that both the a- and b-wave amplitudes were decreased in the LE-with-vehicle-treatment group compared with the control (no LE group treated with vehicle). However, in the LE-with-4-PBA-treatment group, the impairment was significantly attenuated in all stimulation conditions compared to the LE-with-vehicle-treatment group, indicating that 4-PBA preserved visual function after LE (Figure 1A,B,D). Implicit times were preserved, consistent with previous findings [12,15,35,38,39,40] (Figure 1C,E).

### 3.2. 4-PBA Suppressed Light-Induced Photoreceptor Histological Changes and Degeneration

**Figure 2:**
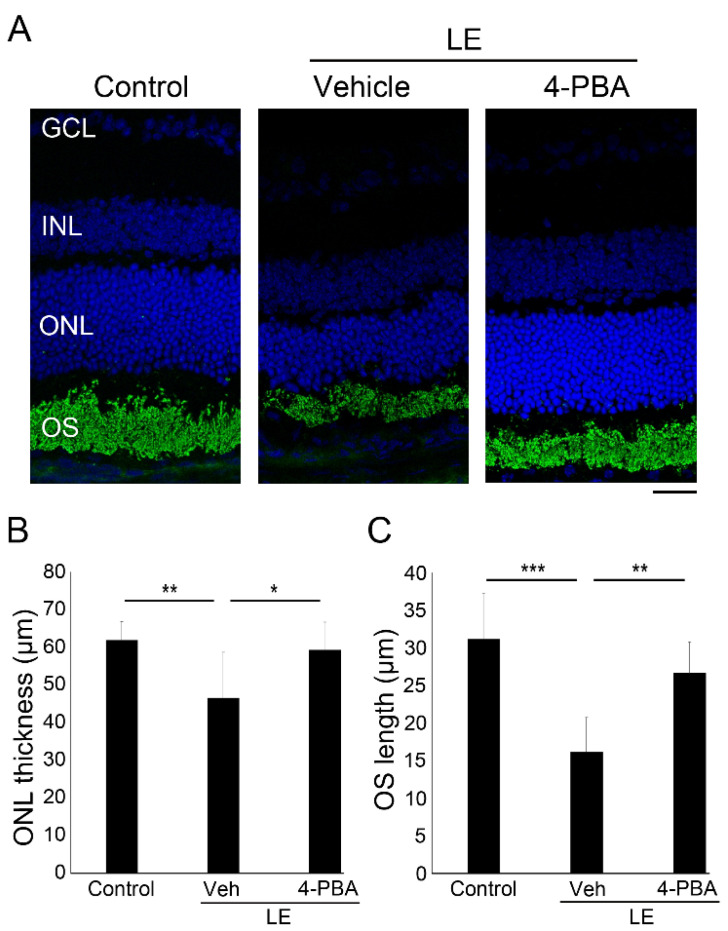
4-PBA suppressed light-induced photoreceptor histological changes and degeneration. (**A**) Representative images of immunohistochemistry for rhodopsin (green) counterstained with 4′,6-diamidino-2-phenylindole (DAPI) (blue) 4 days after light exposure with or without 4-PBA treatment. Reduction in ONL thickness (**B**) and OS length (**C**) were suppressed by 4-PBA. LE, light exposure; Veh, vehicle; GCL, ganglion cell layer; INL, inner nuclear layer; ONL, outer nuclear layer; OS, outer segment. Data are presented as mean ± standard deviation. *n* = 8 in each group. Scale bar, 25 μm. * *p* < 0.05, ** p < 0.01, *** p < 0.001. ANOVA with Tukey post-hoc tests.

We also evaluated the effect of 4-PBA on retinal histological changes 4 days after LE in the eyes used to obtain the ERG data (Figure 2A–C). The LE-with-vehicle-treatment group exhibited a significantly thinner ONL than the control group. Moreover, the length of rod OSs, where rhodopsin is concentrated, was also reduced in the LE-with-vehicle-treatment group compared with control group. However, these changes were significantly suppressed in the LE-with-4-PBA-treatment group, indicating that 4-PBA protected the retina from photoreceptor degeneration induced by LE.

### 3.3. 4-PBA Attenuated Photoreceptor Death after Light Exposure

**Figure 3:**
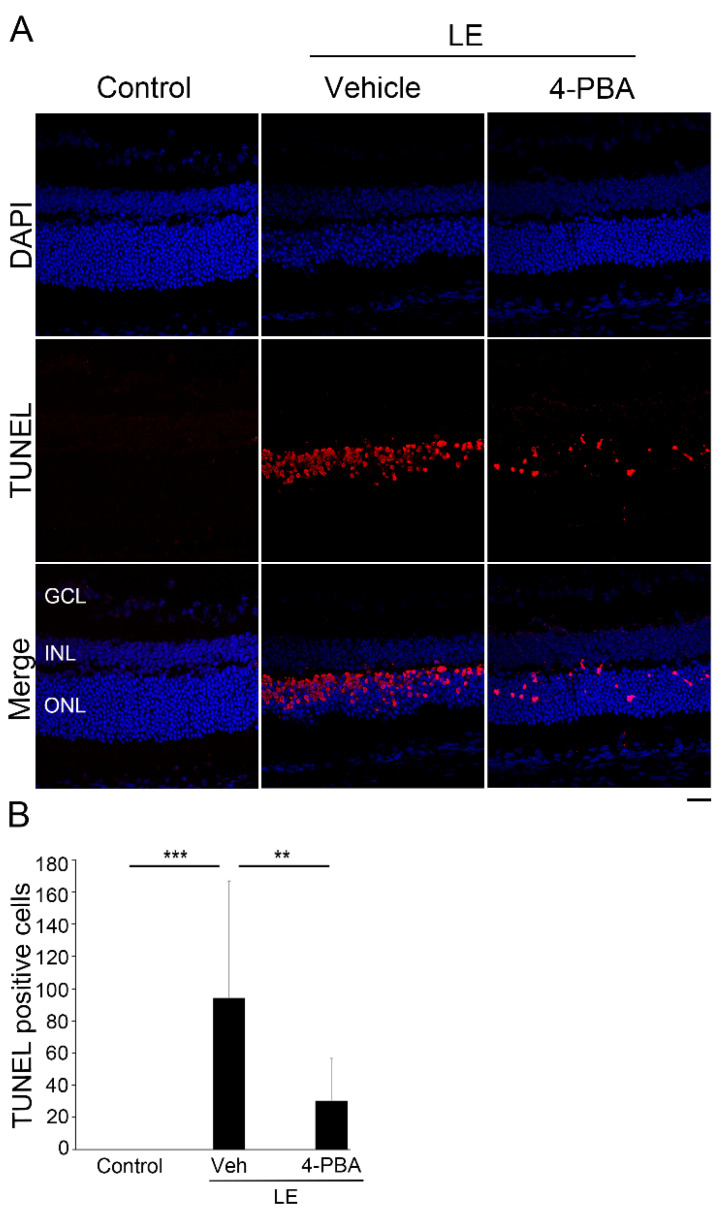
4-PBA attenuated photoreceptor death after light exposure. (**A**) Representative images of the TUNEL assay 2 days after light exposure with or without 4-PBA treatment. Apoptotic cells (red) were only observed in the ONL, i.e., the photoreceptor cell layer. Nuclei were counter-stained with DAPI (blue). (**B**) Number of Terminal Deoxynucleotidyl Transferase2´-Deoxyuridine, 5´-Triphosphate (dUTP) Nick End Labeling (TUNEL)-positive cells in a section, indicating that 4-PBA attenuated apoptotic cell death; there was no significant difference between control and LE-with-4-PBA-treatment groups (*p* = 0.25). LE, light exposure; Veh, vehicle; GCL, ganglion cell layer; INL, inner nuclear layer; ONL, outer nuclear layer. Data are presented as mean ± standard deviation. Control: *n* = 12, LE with vehicle: *n* = 12 and LE with 4-PBA: *n* = 11. Scale bar, 25 μm. ** *p* < 0.01, *** *p* < 0.001. ANOVA with Tukey post-hoc tests.

We further analyzed the number of apoptotic cells using a TUNEL assay 2 days after LE (Figure 3A,B), as this is the timepoint where apoptotic cells become significantly increased in the light-exposed retina [12,15,35,38,39,40]. TUNEL-positive and apoptotic cells were increased only in the ONL, i.e., the photoreceptor layer after LE. However, the apoptotic cells were significantly reduced in the LE-with-4-PBA-treatment group, indicating that 4-PBA treatment attenuated light-induced photoreceptor apoptosis.

### 3.4. 4-PBA Suppressed ER and Oxidative Stress, and Apoptotic Markers

**Figure 4:**
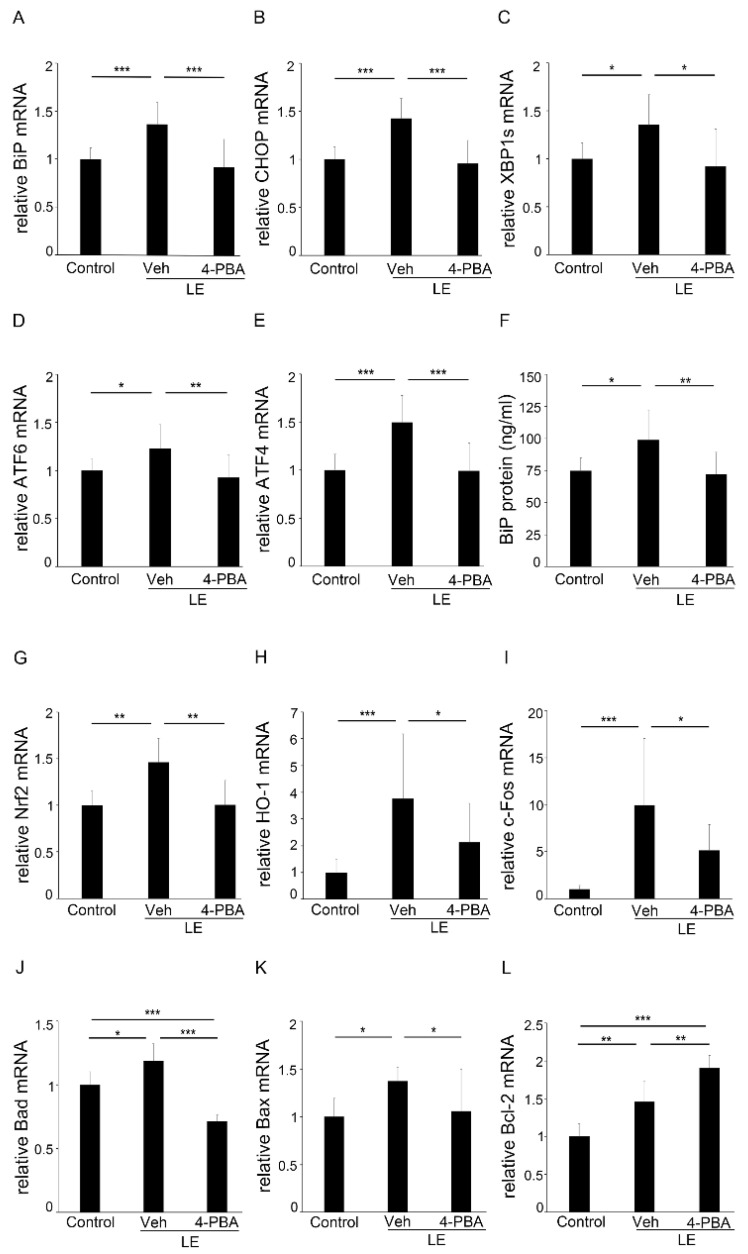
4-PBA reduced ER and oxidative stress, and apoptotic markers. (**A**–**E**) mRNA expression of ER stress markers using RT-PCR 3 h after LE. mRNAs of (**A**) the immunoglobulin heavy chain binding protein (BiP), (**B**) CHOP, (**C**) XBP1s, (**D**) ATF6, and (**E**) ATF4 were upregulated in the LE-with-vehicle-treatment-group, but the mRNA levels remained close to those of the control in the LE-treated-with-4-PBA group. (**F**) BiP protein levels measured using enzyme-linked immunosorbent assay 3 h after LE showed similar results to those of the BiP mRNA levels. mRNAs of oxidative stress markers, (**G**) Nrf2 and (**H**) HO-1 measured with reverse transcription-polymerase chain reaction (RT-PCR )were also upregulated 3 h after LE; however, the levels were reduced by 4-PBA (**I**) c-Fos (3 h), which contributes to photoreceptor apoptosis after LE, and mitochondrial pro-apoptotic factors Bad) (3 h) (**J**) and Bax (6 h) (**K**) were also upregulated after LE; however, 4-PBA treatment suppressed these levels. Mitochondrial anti-apoptotic factor Bcl-2 (**L**) was upregulated immediately (0 h) after LE and was further increased in the 4-PBA-treated group. ER, endoplasmic reticulum; LE, light exposure; Veh, vehicle. Data are presented as mean ± standard deviation. (**A–F**) *n* = 9–12; (**G**) *n* = 6 in each group; (**H**) Control: *n* = 18, LE with vehicle: *n* = 15 and LE with 4-PBA: *n* = 14; (**J, L**) Control: *n* = 6, LE with vehicle: *n* = 5 and LE with 4-PBA: *n* = 6; (**K**) *n* = 11/group. * p < 0.05, ** p < 0.01, *** p < 0.001. ANOVA with Tukey post-hoc tests.

To understand the mechanisms involved in the protective effects of 4-PBA, we analyzed the retinal samples obtained before the light-induced histological changes became evident. We found that the mRNA levels of BiP (Figure 4A), CHOP (Figure 4B), XBP1s (Figure 4C), ATF6 (Figure 4D), and ATF4 (Figure 4E) were upregulated, together with the protein level of BiP (Figure 4F) in the light-exposed retina at 3 h; however, 4-PBA treatment substantially suppressed the ER stress markers (Figure 4A–F), indicating that ER stress was rapidly induced in the retina after LE; however, early treatment with 4-PBA relieved ER stress.

Since light-induced retinal degeneration is related to oxidative stress [12,15,38,40], we assessed the oxidative stress markers; the retinal mRNA levels of Nrf2 (Figure 4G) and HO-1 (Figure 4H) were upregulated in the LE-with-vehicle-treatment group at 3 h; however, the levels remained close to those of the control in the LE-with-4-PBA-treatment group.

Along with this, the mRNA levels of c-Fos, which is a subunit of transcription factor activator protein-1 (AP-1), which is essential for a light-induced apoptotic pathway in photoreceptors [39,40,41], were upregulated in the light-exposed retina at 3 h; however, the levels were attenuated in the 4-PBA-treated group (Figure 4I).

The retinal mRNA levels of Bad at 3 h (Figure 4J), and Bax at 6 h (Figure 4K), were upregulated in the LE-with-vehicle-treatment group; however, they remained at the normal levels in the LE-with-4-PBA-treatment group. Interestingly, expression of Bcl-2 (Figure 4L), which inhibits apoptosis by binding and inactivating Bax [42], was upregulated immediately (0 h) after LE and further upregulated in the LE-with-4-PBA-treatment group.

### 3.5. 4-PBA Attenuates Neuroinflammation

**Figure 5:**
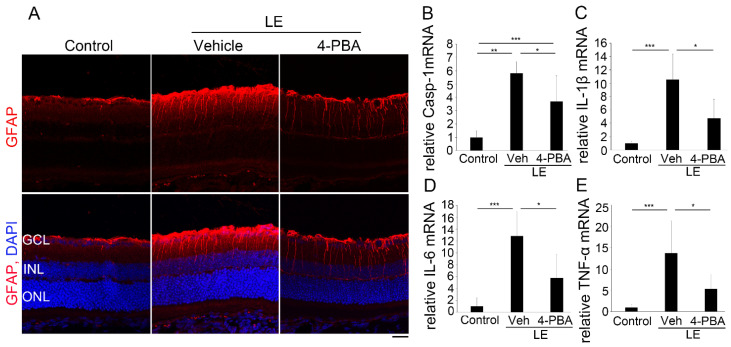
4-PBA suppressed neuroinflammation. (**A**) Immunohistochemistry for GFAP (red) 24 h after light exposure. Light-induced increase in GFAP expression was attenuated by 4-PBA administration. DAPI staining (blue). (**B**–**E**) mRNA levels of pro-inflammatory molecules using real-time transcription polymerase chain reaction at 24 h after LE. (**B**) The Caspase-1, (**C**) IL-1β, (**D**) IL-6, and (**E**) TNF-α mRNAs were upregulated after LE; however, the levels were attenuated in the LE-treated-with-4-PBA group. LE, light exposure; Veh, vehicle. (**B**–**E**) Data are presented as mean ± standard deviation. A statistically significant difference between Control and LE-with-4-PBA-treatment groups was only found in caspase-1 mRNA levels. (**A**) *n* = 4 in each group; (**B**–**E**) *n* = 5–6. Scale bar, 50 μm. * p < 0.05, ** p < 0.01, *** p < 0.001. ANOVA with Tukey post-hoc tests.

Under normal conditions, GFAP is only present at the end-foot of Müller glial cells, and when they become reactive by stimuli such as light damage [38], GFAP expression is upregulated [43,44,45,46]. We found that GFAP expression was induced 24 h after LE; however, it was decreased in the retina of the LE-with-4-PBA-treatment group (Figure 5A).

The mRNA levels of caspase-1 (Figure 5B), which cleaves IL-1β(Figure 5C) to promote its secretion, as well as of other inflammatory cytokines such as IL-6 (Figure 5D) and TNFα(Figure 5E), were also upregulated 24 h after LE. Meanwhile, in the LE-with-4-PBA-treatment group, the levels were comparable to those of the control. Taken together, it was shown that 4-PBA suppressed light-induced neuroinflammation in the retina.

## 4. Discussion

We demonstrated that light-induced visual impairment, retinal degeneration, and photoreceptor apoptosis were considerably suppressed by the administration of 4-PBA, as shown by the ERG, ONL thickness, OS length, and TUNEL assay findings, respectively (Figure 1, Figure 2 and Figure 3). In parallel with ER stress markers, oxidative stress and apoptosis markers were also suppressed soon after LE (Figure 4). Moreover, following neuroinflammation, as seen with induction of GFAP and inflammatory molecules after LE, were also attenuated by 4-PBA treatment (Figure 5).

The a-wave amplitudes decreased 4 days after LE, indicating that photoreceptor function was impaired, consistent with the findings of previous reports [12,15,35,38,39]. The b-wave amplitudes also decreased, which was most likely related to the reduced electric signals from the damaged photoreceptors. Light-induced photoreceptor damages were identified histologically; ONL thinning, which represents photoreceptor loss, and OS shortening, which represents structural damage in the remaining photoreceptors, were obvious at the same timepoints. Notably, all these changes, including functional and histological changes, were clearly attenuated by 4-PBA administration, indicating that 4-PBA contributed to photoreceptor protection. Moreover, apoptotic changes in the ONL preceded, at 2 days after LE, suggesting that photoreceptor loss was mediated by the apoptotic pathway, with apoptotic cells being present only in the photoreceptor layer, consistent with previous findings [12,15,35,38,39,40]. Administration of 4-PBA decreased the number of apoptotic photoreceptor cells, which explained the preservation of ONL thickness found in the light-exposed group that was treated with 4-PBA. While there may have been photoreceptors which finally died after delayed cell death in the current protocol of the study, these data indicated that 4-PBA, at least in part, promoted photoreceptor survival under conditions of intense photo-stress.

In the process of ER stress, accumulation of misfolded proteins in the ER lumen triggers the UPR [12,13,14,23], which is initiated by the dissociation of BiP, also known as GPR78, from the ER transducers PKR-like endoplasmic reticulum kinase (PERK), inositol-requiring enzyme 1 (IRE1), and ATF6, to bind to misfolded proteins to correct the folding [13,17,18,19,20]. Upon activation, IRE1, PERK, and ATF6 initiate the intracellular signal transduction pathways that enhance ER function; however, if the UPR cannot restore ER homeostasis, the same pathways may promote cell death by inducing CHOP and activating the intrinsic apoptosis machinery also known as the mitochondrial apoptotic pathway [13,17,18,19,20].

We found that BiP was upregulated 3 h after LE, both at the mRNA and protein levels as shown by RT-PCR and ELISA, indicating that photo-stress induced ER stress. A high Ca^2+^ level in the ER lumen is essential for protein folding [47,48], and depletion of Ca^2+^ stores in the ER lumen can induce ER stress [48]; because intense light causes an increase in intracellular calcium [11,49], it might have disorganized Ca^2+^ metabolism in the ER. However, in the LE-with-4-PBA-treatment group, the BiP mRNA and protein levels remained similar to those of the control, indicating that 4-PBA was able to suppress induction of ER stress from the beginning. Several studies have suggested that 4-PBA acts as an ER stress inhibitor by aiding in protein folding, assisting with protein trafficking, and preventing protein aggregation within the ER [24,25]. Whether 4-PBA administration had suppressed the UPR by acting on misfolded proteins in the light-exposed retina would be the future topic.

Previous studies have shown that in RP, misfolded rhodopsin has been shown to trigger the IRE1 branch of the UPR and that ER stress may induce photoreceptor cell death in light-exposed retinas and that this effect is mediated by CHOP [12,13,15,19,35,38,39]. In the current study, the mRNA expression of CHOP, XBP1s, ATF6, and ATF4 was upregulated, suggesting that all arms of the UPR were activated and that the UPR had already switched to promote cell death. This may be related to either ER stress being too severe or the stress being too rapid for compensation by the UPR or a combination of both. Consistent with mRNA suppression of BiP, CHOP, XBP1s, ATF6, and ATF4 in the LE-with-4-PBA-treated group, 4-PBA promoted photoreceptor survival and protection.

The crosstalk between ER stress and oxidative stress is involved in many physiological and pathological conditions. In a stressed ER, dysregulation of the formation and breakage of disulfide bond may result in accumulation of reactive oxygen species (ROS) and oxidative stress [50]. An oxidizing environment due to ROS accumulation can further lead to abnormal protein misfolding, which hyperactivates ER stress. Alternatively, the ER and mitochondria are physically and functionally connected through mitochondrial-associated ER membranes, which regulate the uptake of Ca^2+^ released from the ER into the mitochondria; the ER–mitochondrial relationship can stimulate mitochondrial metabolism and ROS production. CHOP-regulated expression of ER oxidoreductase 1 alpha (ERO1α) can stimulate inositol-1,4,5-trisphosphate receptor-mediated Ca^2+^ release from the ER [50,51,52], which may induce ER–mitochondrial calcium flux and further promote ER stress [53]. All these mechanisms together may have created a vicious cycle of ER stress and oxidative stress [22,50,51,52]. In response to ER stress, PERK can phosphorylate and activate transcription factor Nrf2, which regulates the expression of HO-1 to reduce oxidative stress [54,55]. We showed that the levels of Nrf2 and HO-1, both upregulated in the light-exposed retina, remained close to normal in the LE-with-4-PBA-treatment group, suggesting that the crosstalk may have been attenuated by 4-PBA administration, suppressing ER stress.

It is well established that c-Fos is essential for a specific light-induced pre-mitochondrial apoptosis pathway mediated by transcription factor AP-1 in photoreceptors [39,40,41]. Expectedly, we evidenced that mRNA expression was upregulated after LE, consistent with previous findings [39,40]. However, this was not observed in the 4-PBA-treated group. In line with this, expression of Bax, which is a pro-apoptotic effector of the Bcl-2 protein family that controls apoptosis by governing mitochondrial outer membrane permeabilization [56], was upregulated after LE and suppressed by 4-PBA, suggesting that a mitochondrial-apoptosis pathway was activated after LE, but the activation was suppressed by 4-PBA, most likely through suppressing ER stress. This is consistent with the fact that Bax can be found in the downstream pathways of either ATF4/CHOP or IRE1 [55]. Interestingly, expression of Bcl-2, which is an anti-apoptotic protein that regulates mitochondrial dynamics and inhibits Bax [57], was found to be upregulated with LE and further upregulated by the 4-PBA treatment. Since Bad, which is a sensitizer member of the BH3-only proteins that binds to the anti-apoptotic Bcl-2 proteins and thereby indirectly activates Bax [56,58], was upregulated and the photoreceptors substantially died at the later time points, increased Bcl-2 after LE may have been insufficient, and did not compensate for the apoptotic signals in the vehicle-treated group. This was found immediately after LE, when ER stress markers were still found to have no significant difference (Figure S1), proposing the possibility that 4-PBA may act directly on Bcl-2 expression. However, the regulatory network of the Bcl-2 family members is complex and unclear [58], and the mechanism involved in 4-PBA action, either directly or indirectly, on Bcl-2 would need to be further evaluated.

Oxidative stress [8] as well as ER stress [50,51] are closely related to inflammation [8,22,50,51,59]. We found that GFAP was induced after LE, consistent with our previous findings [38], and was attenuated by 4-PBA treatment. Moreover, pro-inflammatory cytokines IL-1β, IL-6, and TNFα, as well as caspase-1, which cleaves IL-1β, were upregulated after LE and suppressed by 4-PBA administration to levels comparable to those in the control; GFAP is expressed in the Müller glial cells, and outside the photoreceptors, impairment of b-wave amplitudes after LE may have been influenced by retinal neuroinflammation induced as a result of photo-stress, although further study is required.

## 5. Conclusions

In summary, we found that early reduction of ER stress in photo-stressed retinas by 4-PBA clearly suppressed visual impairment, photoreceptor apoptosis and loss, and degeneration and was related to suppression of retinal oxidative stress and neuroinflammation. Therefore, the current study will further facilitate the elucidation of photo-stress pathogenesis and will aid in the development of 4-PBA as a therapeutic approach for suppressing progression of retinal degeneration and protecting visual function against the effects of photo-stress.

## Figures and Tables

**Table 1:** Primer list for real time PCR.

Gene	Forward Primer	Reverse Primer	GenBank Accession No.
Immunoglobulin heavy chain binding protein (BiP)	TGCAGCAGGACATCAAGTTC	TTTCTTCTGGGGCAAATGTC	NM_001163434
C/EBP-Homologous Protein (CHOP)	CTGGAAGCCTGGTATGAGGA	GGACGCAGGGTCAAGAGTAG	NM_007837
spliced- X-Box Binding Protein 1 (XBP1s)	CTGAGTCCGCAGCAGGTG	TGCCCAAAAGGATATCAGACT	NM_001271730
Activating Transcription Factor 6 (ATF6)	GGACGAGGTGGTGTCAGAG	GACAGCTCTTCGCTTTGGAC	NM_001081304
Activating Transcription Factor 4 (ATF4)	GAAACCTCATGGGTTCTCCA	TCCATTTTCTCCAACATCCA	NM_009716
Nuclear factor erythroid 2–related factor 2 (Nrf2)	TGGCAGGAGCTATTTTCCATTC	TGCTGTCCATTTCTGTCAGTGTG	NM_010902
Heme Oxygenase 1 (HO-1)	ACGCATATACCCGCTACCTG	CCAGAGTGTTCATTCGAGCA	NM_010442
c-Fos	ATGGGCTCTCCTGTCAACAC	ACGGAGGAGACCAGAGTGG	NM_010234
B-cell lymphoma 2 apoptosis regulator (Bcl-2)-associated death promoter (Bad)	GGAGCAACATTCATCAGCAG	GTACGAACTGTGGCGACTCC	NM_007522
Caspase-1	AGGAGGACATCCTTCATCCTC	CTTGAGGGTCCCAGTCAGTC	NM_009807
Interleukin-1 beta (IL1-β)	AGCTCTCCACCTCAATGGAC	AGGCCACAGGTATTTTGTCG	NM_008361
Interleukin-6 (IL-6)	GAGGATACCACTCCCAACAGACC	AAGTGCATCATCGTTGTTCATACA	NM_031168
Tumor Necrosis Factor alpha (TNFα)	GCCACCACGCTCTTCTGTCTA	GATGAGAGGGAGGCCATTTG	NM_013693
18s	AGCATTTGCCAAGAATGTTTTCA	CCAGTCGGCATCGTTTATGG	NR_003278

## Data Availability

Data is contained within the article or Supplementary Material.

## Supplementary Materials

The following are available online at https://www.mdpi.com/article/10.3390/antiox10071147/s1, Figure S1. ER stress markers immediately (0 h) after LE.
