# Supplementary material for: Neuroprotective Effect of 4-Phenylbutyric Acid against Photo-Stress in the Retina
Source: Antioxidants (Basel). 2021 Jul 20;10(7):1147. doi: 10.3390/antiox10071147 (PMC8301054; doi:10.3390/antiox10071147)
Supplement: Supplementary file 1 [file antioxidants-10-01147-s001.zip › antioxidants-1284889-supplementary.pdf]

Figure S1

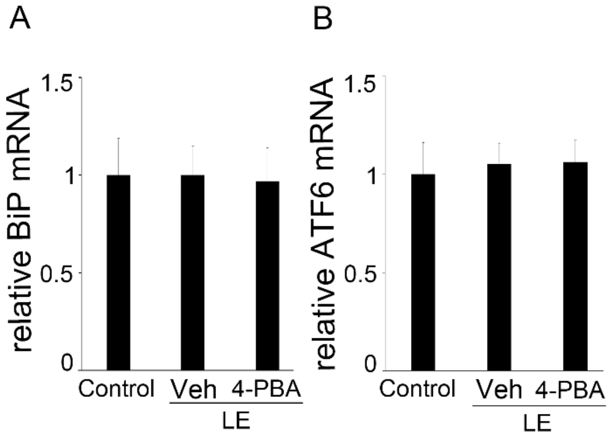

Figure S1. ER stress markers immediately (0 h) after LE. mRNA expression of ER stress markers (**A**) BiP and (**B**) ATF6 using RT-PCR at immediately (0 h) after LE, did not show any significance difference between groups, meaning that the UPR was still not induced by ER stress at this time point. LE, light exposure; Veh, vehicle. Control: n = 5, LE with vehicle: n = 6 and LE with 4-PBA: n = 6. One-way analysis of variance (ANOVA) with Tukey post-hoc tests.
